# The fragile process of Homecoming - Young women in recovery from severe ME/CFS

**DOI:** 10.1080/17482631.2022.2146244

**Published:** 2022-11-11

**Authors:** Silje Helen Krabbe, Karen Synne Groven, Wenche Schrøder Bjorbækmo, Unni Sveen, Anne Marit Mengshoel

**Affiliations:** a Faculty of Health Sciences, Department of Physiotherapy, Oslo Metropolitan University, Oslo, Norway; b Children’s Surgical Department, Division of Head, Neck and Reconstructive Surgery, Pediatric Nurse, Oslo University Hospital, Oslo, Norway; c Faculty of Health Studies, VID Specialized University, Oslo, Norway; d Faculty of Health Sciences, Department of Occupational Therapy, Prosthetics and Orthotics, Oslo Metropolitan University, Oslo, Norway; e Department of Physical Medicine & Rehabilitation, Occupational Therapist, Oslo University Hospital, Oslo, Norway; f Department for Interdisciplinary Health Sciences, Faculty of Medicine, University of Oslo, Oslo, Norway

**Keywords:** Adolescents, children, women, chronic fatigue syndrome, myalgic encephalomyelitis, recovery, lived experiences, narratives

## Abstract

**Purpose:**

To explore the recovery narratives of 13 young women who had fallen ill with severe Myalgic Encephalomyelitis (ME), also known as Chronic Fatigue Syndrome (CFS), during childhood and adolescence, with the focus on what they had to say about their past experiences from the perspective of the present.

**Method:**

A qualitative narrative approach, informed by a phenomenological theoretical perspective, was adopted to explore what the women found significant and meaningful in their recovery process. Data analysis of in-depth narrative interviews was performed which are presented to readers through the stories of two particular participants.

**Results:**

The first story describes how one participant made a recovery by testing her body’s tolerance and working to create a more confident self. The second story describes a complex exploration of possibilities for action in recovery, along with a struggle to make sense of setbacks and hold on to what has been gained.

**Conclusion:**

Recovering from ME/CFS emerges as an inter-personal, contextual, fragile and nonlinear process of homecoming, based on gradually rising bodily based self-knowledge. Illness slowly fades away into the background, and there is the prospect of a healthier tomorrow.

## Introduction

In this article we present findings from research involving young women who experienced severe Myalgic Encephalomyelitis (ME), also known as Chronic Fatigue Syndrome (CFS), during childhood and adolescence but who had subsequently recovered, either fully or partly.

Our study forms part of a larger Norwegian project exploring young girls’ and female adolescents’ experiences of falling ill, being ill and being in recovery from ME/CFS.

ME/CFS is a serious and complex illness characterized by debilitating fatigue, post-exertional malaise (PEM) and loss of physical and cognitive function (Rowe et al., 2017). It can affect persons of all ages, cultures, ethnic groups and socio-economic groups (Jason et al., 2020), although women are overrepresented (Ranjith, 2005). To date, the aetiology of ME/CFS has not been established and no curative treatment for the illness has emerged (Sharpe & Greco, 2019). There is no objective clinical test to verify the illness.

From a health care perspective, children and adolescents are generally considered to have a better prognosis for recovery from ME/CFS than adults (Moore et al., 2021; Rowe et al., 2017). However, while for adults there are multiple ways of defining and measuring recovery from ME/CFS (Adamowicz et al., 2014), recovery rates among children and adolescents vary considerably, and there is no international consensus on how to measure and define recovery (Moore et al., 2021). The most common outcome parameters for children and adolescents relate to school attendance, fatigue, and improvement in physical functioning (Moore et al., 2021).

The complexity of the illness, together with its fluctuating symptoms, and their tendency to vary over the course of an individual’s lifespan, make it difficult to judge whether someone is in recovery or is simply experiencing a temporary, random improvement (Devendorf et al., 2019). Acquiring longitudinal data on those recovering from ME/CFS represents a further challenge. Persons who recover, particularly at an early age, may stop seeking medical help, without physicians knowing whether the condition of such patients has actually improved. The fact that a patient has recovered will not necessarily be registered and the physician will not have the opportunity to include the patient in medical research (Devendorf et al., 2019).

The lack of international consensus on how to define recovery from ME/CFS also makes it difficult to differentiate between those in the process of recovery and those who regard themselves recovered from ME/CFS. In previous research, children and adolescents with ME/CFS have revealed very different perceptions of recovery, and have often found it difficult to describe what recovery involves (Harland et al., 2019). One reason for this lack of clarity is that when children and adolescents are ill for long periods, illness may become the “new normal”, making it difficult to consider what complete recovery might mean (Harland et al., 2019).

Given the complexity of ME/CFS, and the fluctuating character and intensity of symptoms over time (Parslow et al., 2018; Rowe et al., 2017), a child or adolescent patient can experience improvement in some symptoms while others remain unchanged.

With two exceptions (Harland et al., 2019; Jelbert et al., 2010), few studies have examined children’s and adolescents’ experiences of recovery (Moore et al., 2021). Harland et al. (2019) found essential elements of recovery to include resuming meaningful activities with peers, avoiding setbacks and enjoying flexibility in life. Jelbert et al. (2010) found that adolescents experienced personal growth, including increased confidence and maturity, as a result of their illness and recovery. Their illness experience also strengthened their relationships with family and friends. However, returning to everyday life and being with others was also experienced as difficult and scary, especially at a time when adolescents were facing the challenge of transitioning from being ill to getting better (Jelbert et al., 2010).

Despite such research, there remains a gap in the literature regarding what the process of recovery means and entails for young individuals with ME/CFS. What nuances and variations do their experiences reveal in terms of bodily changes and everyday life? Given the lack of consensus on what constitutes recovery in children and adolescents, there is a need to focus on this process as narrated and experienced by those who have first-hand knowledge of it (Moore et al., 2021).

For our study, we gathered and explored narratives told by young women in recovery from ME/CFS who had been severely ill since childhood or adolescence. We sought to identify what they found significant and meaningful in their recovery process by posing the following question:

*What and how do young women previously suffering from ME/CFS during childhood and adolescence tell about their bodily experiences of becoming better from the present position of being in recovery or being recovered?*

## Theoretical framework

In terms of theoretical perspective, our research was inspired and informed by the insights of the French phenomenologist Maurice Merleau-Ponty (1962), in particular his understanding of the lived body as the basic source of all our experiences and perceptions. We argue that a phenomenological perspective can illuminate the lived experiential meanings embedded in individual stories of bodily phenomena: still acknowledging that there are also pre-conscious and embodied meanings at play that are not immediately apparent to the person narrating their story. In our study, we explore young women’s lived experiences of recovery from ME/CFS as narrated in retrospect, focusing especially on bodily changes and everyday life. For Merleau-Ponty (1962), humans are at once individual, relational and situated bodily beings. Here, the body, far from being the passive object of our consciousness, is understood as an inseparable, interpersonal, sensitive, interpretive and significant embodied and embedded lived body (Merleau-Ponty, 1962). Our existence is a situated, inter-subjective co-existence: we see and are seen, we hear and are heard, and so on. As Merleau-Ponty (1962) explains, this interactive bodily existence means that we both are and have our body—we are always both subject and object, never either-or. In line with Merleau-Ponty (1962), we argue that it is through their bodies that the young women in our study perceive themselves and their surroundings, express themselves and communicate with others.

Our study is also influenced by ideas developed by Fredrik Svenaeus (2001), himself inspired by the German phenomenologist Heidegger, regarding experiences of being-in-the-world during illness. When people fall ill, Svenaeus (2001) argues, they experience a constant sense of obtrusive unhomelikeness in their being-in-the-world (Svenaeus, 2000, 2001). This can involve feeling disorientated, helpless and despairing, in contrast to finding oneself “at home” in one’s being-in-the-world. Such feelings contrast with the parallel notion of “homelikeness”: the normal, everyday flow of life to which we rarely pay explicit attention to (Svenaeus, 2000). The experience of recovering can be understood as slowly regaining or coming to terms with a more homelike being-in-the-world. While this may not necessarily mean a return to the old, pre-illness homelike being-in-the-world, it involves the individual actively exploring and discovering new ways of being-in the world, towards creating a new “homelikeness”.

In our study, we sought to explore recovery as a process that changes over time. For this reason, we applied a narrative methodological approach. Since storytelling is understood as our fundamental way of making sense of our experiences (Polkinghorne, 1988), we argue that retelling the story of their illness and recovery offers the young women in our study a way of “repairing” their biography as well as involving them in a profound meaning-making process (Riessman, 2008).

When retrospectively making sense of recovery in relation to the past, present and with expectations for the future, we do so in a particular way: we structure our experiences in the form of stories (Mattingly, 1998). Storytelling is a meaning-making process, where events are linked together in a temporal way to create a coherent whole. The perspectives of those who have recovered, or are in recovery, from ME/CFS, are unique and valuable. Such individuals are in a position to narrate coherent stories and give meaning to their former experiences by connecting the past with the present and onto a possible future.

Mattingly and Lawlor’s (2001) descriptions of “healing dramas”, where moments stand out as significant for the person, can contribute to an understanding of recovery as an inter-personal process. Within this understanding, recovery might not be about returning to the healthy body one once was but rather about reclaiming the body and exploring the body-related possibilities that are now conceivable (Mattingly & Lawlor, 2001). Viewed from the perspective of being in recovery, these could be healing moments — although ones that might remain invisible to others (Mattingly & Lawlor, 2001).

In this study, then, we combine a phenomenological theoretical perspective with a narrative approach to explore and comprehend the lived experiences of recovery over time.

### Methods

#### Recruitment and participants

Participants for this study were required to be women aged between 16 and 30 who had fallen ill with ME/CFS during childhood or adolescence. Women were chosen because of the higher incidence of ME/CFS among females. The 16–30 age range was chosen in order to recruit participants who had been ill during childhood and adolescence but who were currently considering themselves to be in partial or full recovery from ME/CFS. Participants had to have been diagnosed with ME/CFS by a physician, to have been ill for more than a year, and to now consider themselves to be considerably better or without symptoms. They were also required to speak, read and write Norwegian fluently.

We recruited participants by placing an advertisement on two Norwegian websites concerned with women’s health care and rights in society. Potential participants contacted the first author directly by phone or email, and those who met the criteria received detailed information about the study and a consent letter by email. A total of 17 women made contact, of whom only 13 took part in the study. Of the four women who did not participate, three decided not to take part in the study and one did not meet the criteria (she had not been diagnosed with ME/CFS by a physician). The interviews were conducted shortly after each participant made contact with the first author.

Our 13 participants were aged between 16 and 29 years (median age 24). Three of them had fallen ill before the age of twelve; nine had fallen ill during adolescence; and one had become ill in late adolescence/early adulthood. While some considered themselves to be in significant recovery, most considered themselves to be fully recovered from ME/CFS. At the time of the interviews, the women were either engaged in full- or part-time education or were working full- or part-time. Six were married or living with a partner, and one had a child. The women lived in different parts of Norway, both rural and urban, and all described themselves as having grown up in ‘middle class’s homes. None of the participants was previously known to the first author.

Prior to the interviews, participants were informed about the aims of the study and what their participation involved. They were told that they would be interviewed by a female paediatric nurse with extensive experience of clinical work with children and adolescents.

#### Narrative interviews

Interviews were conducted by the first author between May and September 2019, at participants’ homes, the local library or in a university setting. Each interview lasted from 60 to 150 minutes, was digitally audio-recorded and was transcribed verbatim by the first author shortly after its completion.

In line with the recommendations of Riessman (2008), participants were encouraged to speak freely and with minimal interruption about the changes and improvement in their condition they had experienced over time. The first author began by asking open-ended questions such as “Can you tell me about the time before you experienced starting to become better?” Typical follow-up questions included “Can you tell me about how you first became aware there was an improvement in your condition?” and “Can you tell me about the experience of becoming better?”

Throughout the interviews, participants were encouraged to take their time, reflect and rephrase. This gave the first author the chance to follow up unexpected comments or changes of direction. It also facilitated the generation of extended narratives, as described by Riessman (2008), and gave participants space to create meaning and coherence during their storytelling. Having experienced major disruptions to their lives, the women were ready to offer extended, in-depth descriptions, sometimes with unexpected turns (Riessman, 2008). Additionally, encouraging participants to speak freely and retain control may have contributed to some equalization of the power relation in the interview situation.

## Analysis

Data analysis was conducted by the first author in close collaboration with the four co-authors having professional backgrounds of nursing, occupational therapy and physiotherapy and experienced with various qualitative research methods. The narrative analysis was inspired by the work of Riessman (2008), specifically her thematic and structural way of analysing narratives, and by Mattingly’s (1998) concept of healing dramas and telling moments. Throughout, our focus was on the events participants had found especially meaningful during their process of becoming better. In line with Mattingly (1998), we understand events as telling moments, ones when individuals understand themselves and their situation in new ways, enabling them to take new forms of action.

First, the audio-recorded interviews were listened to several times. This was followed by a close reading of each interview to get an overall impression of what the narrative was about.

In line with Riessman (2008), what was told was analysed with narrative thematic analysis. At this stage we posed the following analytic questions:

- What are the young women telling about the experience of being in recovery?
- What kind of telling moments or events emerge from their accounts?

In our narrative structural analysis, the focus was on the temporal structure of each story: its beginning, middle and end (Riessman, 2008). Furthermore, how the stories were told entailed organizing participants’ events seeing how they were interconnected and trying to reach a meaningful whole (Mattingly, 1998). By comparing and contrasting across the analysed interviews, we gradually enabled to develop two storylines. During this process of analysis, we found differences in how the recovery process was narrated, which is illustrated in the two storylines. While some stories involved events that were ordered temporally (making the narrative easy to follow), other stories included elements that were more challenging to comprehend. This tended to be the case with stories that seemed incomplete or tentative, often told in a hesitant way. Events representing telling moments (whether ordinary events that were suddenly experienced in new ways, or totally unexpected events) were coded and further explored (Mattingly, 1998). The stories, while various and unique, shared some common patterns and themes.

In line with the narrative tradition (Riessman, 2008), we present our in-depth analysis of two such stories, which we regard as especially nuanced and illuminating. At the same time, although different in details, these storylines are also seen in the stories of the other participants. The first storyline, a structured, finished, and coherent piece of storytelling, contrasts with the second, which presents a more unfinished, not fully developed storyline. The two storylines, however, share a common plot: struggling from being “unhomelike” to gradually becoming a homelike being-in-the-world. We argue that this narrative plot provides one possible way of understanding what a personal recovery process from ME/CFS can be like.

### Ethical considerations

The study was approved by the Norwegian Regional Committee for Medical and Health Research Ethics, Social Science Data Service, and conducted in accordance with the Helsinki Declaration Act and Health Research Act of Norway. The Service for Sensitive Data was used as required for collecting and storing sensitive data. Only the main supervisor (KSG) and the first author have had full access to the interviews and the transcribed material; the three other authors have had access only to excerpts from the anonymized transcribed material. Informed consent was given by the participants prior to each interview.

## Findings

Eight out of our thirteen young women participants considered themselves to be completely recovered from ME/CFS and were either working or studying full-time or planning to work or study full-time at the time of the interview. The remaining five considered themselves to be much better, but still to some extent experiencing fatigue and aware of the need to get enough rest. All the women described how they had a greater need for structure and routine in their daily life (for example, in relation to meals and sleep) than they had experienced prior to illness. All told of how, when they fell ill, their parents had sought different kinds of treatment for them, and how they themselves tried different treatments in the hope of getting better.

When it came to how they told their stories, however, participants varied considerably. Some of those who had recently recovered or had a sudden recovery told their stories by jumping back and forth in time and adding remembered details along the way. This was storytelling that was a bit difficult for the interviewer to follow; as researchers, we understand it as an unstructured form of storytelling. Others told their stories by describing their current situation before moving back in time. But most of the women started by telling about their life prior to illness, then moving on to describe falling ill and eventually becoming better.

In the excerpts from transcripts presented below, all participants appear under pseudonyms. Any quotation that might disclose a particular participant’s identity has been omitted. The stories are written in the present tense, even in relation to past experiences, in an attempt to underline the developing nature of the narratives.

The first story, told by Cornelia, is that of a young woman who considers herself to be fully recovered from ME/CFS. The story is an example of structured, finished, and coherent storytelling: Cornelia begins by describing her life prior to her illness, then moves on to describing her experiences of falling ill and, eventually, becoming better.

The second story is told in a different way. Hedda is a young woman who considers herself to be in the process of recovery from ME/CFS, rather than fully recovered. While her narrative reveals elements of structured storytelling, it appears somewhat unfinished and chaotic when compared with Cornelia’s account. While narrating her story, Hedda jumps back and forth in time as she seeks to make sense of her recovery. This may be related to the fact that she experienced a sudden awakening after a long period of being bedridden with ME/CFS.

While both Cornelia and Hedda recount a long process of returning to everyday life, a struggle to come to terms with a changed and changing body in order to find a way to rebuild one’s body and everyday life, they deploy different storylines.

We characterize the theme of the first storyline as “Exploring and building an understanding of bodily limits and capacities”. Here, a woman who considers herself to be fully recovered from ME/CFS describes a recovery process in which she tests her body’s tolerance and seeks to build a more confident self.

The theme of the second storyline is “Bodily awakening of the self: From a dark place to a fragile and unfinished process of reuniting with one’s own body and world”. While this woman considers herself to be significantly better from ME/CFS, her recovery process remains ongoing. In her narrative, she describes how she wakes up to a world of possibilities for action, after being isolated and severely ill for years.

In the excerpts from transcripts presented below, the symbol […] signifies that two identified sequences were found to be related, while three dots … indicate a pause in the narrative.

### Exploring and building an understanding of own bodily limits and capacities

At the time of her interview, Cornelia is 25 years old. She is about to become a full-time student after being ill for nearly ten years. When she first fell ill, she was diagnosed with Mononucleosis. She never seemed to recover from it, but despite this, she was advised to stay physically active and keep up full-time schooling. It was only after nearly two years of seeking medical help and struggling with exhaustion and malaise that she was finally diagnosed with ME/CFS. During her illness, Cornelia experienced symptoms that fluctuated from moderate to very severe. For a long time, she attempted to keep up with her schoolwork but found doing so took all her energy. In the end she was forced to quit school because of her high absenteeism. She describes her years before recovery as a downward spiral in which her condition always worsened and she suffered “collapses” which left her bedbound, isolated, and severely ill for months. She would get somewhat better, only to collapse again.

During her collapses, Cornelia experienced profound fatigue, diffuse pain, memory loss and loss of appetite. She described how her parents struggled to help her, searching for treatments, and battling to provide her with sufficient nutrition. She went through a variety of treatments, including physical therapy, psychotherapy, diets, and the Lightning Process (a three-day personal training programme developed and trademarked by British osteopath Phil Parker). But while these treatments gave her some tools to cope with her situation, she continued to get worse.

However, during a period of more moderate symptoms when she was able to leave her bed, she made one final attempt to get treatment, at a public health out-patient clinic for people suffering from different kinds of exhaustion disorders. As she explains:

We (the family) understood I had to try something completely different … I couldn’t stand another collapse … My first meeting at the clinic … well, I got a good feeling as if they knew what they were doing. I was together with people of all ages, and everyone got their own exercise plan … we met like four times a week […] There was nothing left of my body, I couldn’t do a thing … but you were supposed to take it very slowly … not overdoing activity, … kind of get in touch with your body … very gently … slow Yoga or Pilates. They helped me to find the level I could do without getting worse the next day.

For the first time in years Cornelia was outside her home, in a safe place with others who somehow understood her situation. Her embodied way of being had changed dramatically during the illness, leaving her with little strength to do anything. Despite this, she was guided to slowly explore her body and possibilities for action. She gained an understanding of how much her body can tolerate without experiencing exhaustion afterwards. This is how Cornelia describes the change she experienced:

It was wonderful to be social again … but man, we were a bounce of tired people (laughs). For the first time in years, I had like a schedule, and I could go through with it […] I focused on my body and movement … stretching … Not pushing myself … It was so nice to find the right level … and wake up the next day with a good feeling … and after some time at that level, slowly challenge myself and do a bit more. I learned to listen to my body … and it went well. I had to learn my new limits and accept them. I was done with pushing myself and this was how I wanted to do it […] You could say this was a turning point for me …

Sharing her experiences with others in a similar situation and meeting on a regular basis prove especially important to Cornelia. Together, they create a kind of supportive community. Exploring what her weak body can achieve, with support from others, is like balancing on a tightrope, with little room for mistakes: she knows that if she overdoes a particular exercise, she will feel exhausted the following day. All the same, she knows she has to test her body so as to understand what she might be capable of. Without that testing, she cannot know what her body can tolerate. Cornelia senses she had started something within herself. By doing these slow movements she is on her way to reconnecting with her body, and it feels good.

In the past, Cornelia had been determined not to show others how ill she was. Additionally, she had pushed herself to attend school, knowing that otherwise she would be forced to quit. But at the clinic she does not need to hide her illness:

It’s always been the social things that have worn me out … and I could never reveal any weaknesses. People never understood I was ill … because I didn’t look ill and when I got worse, I was not at school … at school I continued wearing my “mask” and played the healthy Cornelia … with perfect makeup, a smile and always social … It was exhausting to play that role. After a while I had to quit school because of my absenteeism and I became severely ill … isolated, lost all my friends (swallows). I didn’t understand that I pushed myself beyond […] No one else at the Clinic was wearing “a mask” so I just stopped … was honest with myself … I stopped pretending I was healthy … which saved me a lot of energy.

Despite her illness, Cornelia had tried to conform with social expectations. She sought to present herself as a healthy person and a perfect version of herself, instead of paying attention to her own needs and limits. She adopted a “mask” to fend off questions from others, and continued attending school, telling herself “If I just play healthy, I am healthy”. This situation proved unsustainable, and Cornelia found herself bedridden for months at a time.

A breakthrough is achieved when Cornelia is able to reveal the fact that she is ill in the presence of supportive others with whom she feels safe. Patiently following the advice she is given at the clinic, she now pays attention to her body. At last she can concentrate her energy on getting well. Cornelia attends the clinic for nearly three years. At times she is uncertain whether she is getting any better. But then, something happens which helps her discover how much better she actually has become:

I think it was a year after I started the treatment that we (the family) went to our cabin and everyone wanted to go skiing, I was a bit insecure; should I try? My family didn’t want to push me … but then I went on an 8 km roundtrip on my skis … slow, and my dog pulled me a little but still … such a wonderful experience … a relief … I got tired, but it was a good feeling … For the first time I knew there was a big change … that I had done something right. When things are changing slowly you don’t always see the progress …

The event stands out as a long-awaited confirmation, a quiet moment of understanding and a certainty that she has regained sufficient strength in her body to restart everyday activities. She is heading in the right direction. Tiredness used to be something she feared, but for the first time in years, she experienced tiredness as a positive and natural part of being. Her body used to be experienced as an obstacle, but now she has reclaimed a sense of its possibilities for action. Cornelia reflects on her illness and recovery process thus:

The further I get, the more confident I am … I learned to understand myself … now I know how to handle my illness […] If you want to get well, you need support from others and without my family, I would’t be here … and the folks at the clinic (smile) … and you must make some hard choices, like if a person wears you out, you have’t let go … you must cherish your energy … decide how you would like to spend it […]

I need my routines … the balance between sleep, rest and activity, I need to eat regularly so I need to plan … do things that makes me happy! I can even do things that are socially exhausting for me … like going to a party … I know it costs and I’ll need to rest afterwards … but it’s worth it and I know I`ll be fine. I think I’m as healthy as I could get … from my perspective then … mm … I’m done comparing myself with others … I’ve been ill for more than ten years … of course I wish I hadn’t been ill … but my life took a turn … and I learn to look on the bright side.

With the help of others, Cornelia gains an understanding of what her body can tolerate and how to live with the illness. She still needs to prioritize so as to manage her life but she has learned to embrace the future, avoid dwelling upon the past, focus on the good things in her life, and limit what drains her of energy.

Cornelia’s recovery storyline tells of a growing understanding and awareness of body and self. Left with a vulnerable and strange body by her long-term illness, Cornelia experienced a kind of disconnection from her body. When placed in a safe world shared with others, she slowly begins to explore her body through movement. Aided by significant helpers, she becomes more aware of her bodily self and who she wants to be. Slowly an experience of confidence and belongingness in her own self develops. The illness is slowly fading into the background, and she makes various adjustments. More than ever, however, she is does focusing on her future.

### Bodily awakening of the self: From a dark place to a fragile and unfinished process of reuniting with one’s own body and world

At the time of our interview, Hedda is 27 years old and living in her own apartment. She explains that she had little experience of illness prior to ME/CFS. Unlike Cornelia, she had no infection or other explanation for why she became ill in the first place. She was diagnosed with ME/CFS at the age of twelve after a long period of pain, exhaustion and sensitivity to light and sound. For the next seven years she was severely ill and bedridden most of the time. Eventually she became unable to eat or go to the toilet by herself, spending her days lying completely still in bed in a darkened room.

Looking back, Hedda recalls feeling very fragile but also alert during this period. Her parents did all they could to spare her distress, for even the smallest thing, such as eating or changing her diaper, was painful and stressed her out. She was too ill to receive any kind of treatment. She describes it as a challenging situation for her family: they knew she is suffering but didn’t know how to help her. Naturally, it was an extremely difficult situation for Hedda herself and for a long time she showed no signs of improvement.

From the perspective of today, Hedda describes how severely ill she was:

The last year was quite serious … I could barely be fed via the nasogastric tube (swallows). So nauseous … I didn’t think it was possible to suffer this much and still be alive (pause, looks out the window) time was moving slowly … I wanted to die … I was done living. I think it was hard on my parents […] mm how should I continue … (pause) In the beginning it was difficult for me to talk about it … I could easily start to hyperventilate, it was traumatic … it’s a bit easier now … like telling you about it.

For Hedda, her illness was like living in a kind of borderland, a place of stagnation between life and death. It was as if time had stopped, as if everything in her body had stopped, so that her body could hardly absorb food. Life was transformed into merciless, unbearable and eternal torment. All she wanted was an end to her misery. Even later, during recovery, merely thinking about her illness could easily take over Hedda’s entire being. But today she is able to control the feeling, making it possible for her to share her story.

After years of lying still in a darkened room, Hedda experienced a sudden turning point in her life:

I remember there was a change that summer … small change … I remember I started to move … that I felt like moving my arm and … maybe I turned around … because I had been lying on my back the entire time … I didn’t dare to believe … but from time to time I dreamed about becoming better … I didn’t believe I could get well, but that I could be able do something … but sometimes I didn’t believe there was any improvement at all.

Out of the blue, Hedda experiences an inner urge to move herself. She slowly begins to make small movements, carefully and cautiously trying to reconnect with her body after being totally disconnected from it for a long time. Her small movements make her think and hope about getting better, although such hope is fragile and filled with ambivalence: she lacks any confidence in her body and fears it may fail her. She does not dare to hope too much.

However, Hedda’s parents are more optimistic. Following this event, they invite therapists experienced in the treatment of ME/CFS to their home. Her parents have heard about another girl who had been severely ill with ME/CFS but who is no longer bedridden following treatment of this type. This is how Hedda describes what happened when the therapists arrived at her home:

I was told someone was coming to see me, but my parents didn’t tell me much … then they came and I remember one of the women asked me: “Do you want to get well?” I did not understand … was this an option? … to get well? I was supposed to press her hand if I wanted … it took a long time … but then I pressed her hand.

Hedda finds herself confronting a challenge. After being “sheltered” for years in her unpredictable and traumatic state, she is asked to make a decision. Does she want to get well? Such a prospect is so far away from where she is that it is incomprehensible. She wants to get well, but at the same time she is reluctant to commit herself. She hesitates. What does the question even mean? What does it demand of her? Can it actually mean that she can get well? After Hedda signals she wants to get well, the therapists do something totally unexpected:

They (therapists) drew the curtains and opened the window … helped me so I could sit up in bed … I saw the faces of my family which I hadn’t seen in years. I recall the lovely feeling … and letting the daylight into the room … light used to make me suffer […] The next day they held (supported) me and I tried to walk … mm … and I think I sat outside on the terrace, so human in a way.

By saying “yes”, Hedda enters into an alliance or contract with the therapists, enabling them to take the initiative and begin therapy. It is as if Hedda has woken up from long-term hibernation as she is literally as well as symbolically lifted into the daylight. It is the start of an awakening. Hedda has committed herself to re-entering life beyond her bed and darkened room. There is no turning back. Just sitting up in bed and being able to see her family becomes a dramatic, observable healing moment.

As Hedda starts on the long road to reuniting with her body and the wider world, she experiences joy. But at times she is also overwhelmed by exhaustion, resulting in setbacks:

In the beginning it was such a pleasure waking up in the morning … taking part in life and spending time with others. I recall how others would complain about being tired in the morning, and I just thought ‘it’s fantastic to be able to leave the bed!’ It’s like I’ve heard from others (with ME/CFS), that it was like rising from the grave … to get my life back. I can remember the feeling very well […] I had a physical therapist who helped me, we went for short walks … besides that nothing special … I was surprised how quickly my body rebuilt itself after so long a time … mm … in time I was kind of bored of being home and I started going to lectures at school … I felt very grateful […]

But I had several periods when I felt down and thought ‘this is not going my way’ … I had like setbacks (exhaustion) and became terrified … not that I became totally bedridden, but I remember I had to spend days on the couch … terrified I was falling ill again … think it made me worse. What if I don’t get any better! I was so stressed. The negative thoughts kind of pulled me down again … I had to pause for weeks, maybe months, before I could return to where I had been.

Hedda experiences a tremendous sense of gratitude of being alive. She compares being finally able to leave her bed to a kind of resurrection, using a powerful metaphor, “like rising from the grave”, to emphasize the extraordinary experience. But awakening after severe illness is not easy. Hedda is still fragile; her strange body is recurrently tired or exhausted. All this makes Hedda insecure. What is happening and what is she supposed to do? She fears she is falling ill again, and she does not know how to navigate her way so as to avoid getting worse again. She finds herself repeatedly forced to take a step back and withdraw from those around her.

All her life, Hedda explains, she has battled excessive self-criticism:

I used to be so self-critical, you know, ever since I was a child … it took most of my time and I could never say no to anyone … wore me out … I didn’t understand it at the time … I had to learn to get to know the feeling and understand that it stressed me out […] I remember the first time I failed to use the technique (from the therapist): you know, I tried to picture myself in a comfortable state … tried to relax and told myself nice things … but then I totally lost focus and told myself I couldn’t do it! Same thing, over and over … frustrating … after a while I managed to use the technique to stop the negative thoughts and kind of stop the stress … it’s what I need to do when I kind of lose direction … I could easily end up in the ditch again. At one point it became very clear to me: okay, I am exhausted, but it’s not all about the lack of energy … the only thing I need is actually peace and quiet … I need to be at peace with myself. Then I gain energy and do what I want … and be with others.

Before Hedda fell ill, she was already giving herself a hard time. She spoke of always prioritizing others’ needs and desires over her own. In retrospect, she understands how this may have contributed to her experience of exhaustion. She learns to pay attention to her body so she can better understand her own needs and desires. This enables her to find some balance in her life and reach towards inner peace.

Hedda uses the metaphor of “the ditch” to describe her setbacks: times when she experiences symptoms and loses faith in getting well. She tries to help herself, but it is a lonely and difficult process. Today, Hedda considers herself to be significantly better but still in recovery:

It’s been a long way of rebuilding myself and returning to life (swallows) […] I tried to regain contact with my friends, but I think it was difficult for them … made them uncomfortable … because I had been so ill … I figure they didn’t understand … I was at a totally different place … they were deciding which school to go to, and I was most concerned about rebuilding my legs […] I try to liberate myself; my family means well - but I’m not a child anymore … I must try and fail on my own … otherwise, I will never move on […] My doctor told me to stop comparing myself with others who have never been ill and focus on how far I have come, despite being so ill … I need to compare myself with myself.

No longer sheltered from the outside world, Hedda explores ways of becoming an adult and gaining a new, independent way of being. This involves great effort and it’s a process she is doing on her own. She knows that others cannot possibly understand what she has been through or how ill she actually has been. She must rebuild herself and her life from scratch.

When Hedda enters adulthood, she does so without having passed through the possibilities and experiences that are characteristic of adolescence.

Hedda’s storyline is one of resilience but also of continuing fragility. Awakened from her long, merciless hibernation, Hedda still battles to create an understanding of what she has been through and make meaning out of the experience. Her first experience of hope and recovery was her inner urge to move. Then she received help to understand that there were possibilities waiting for her. Now, finally, she is in the process of reuniting with her body. From the perspective of today she understands recovery as an ongoing, self-driven process that takes time. While she needs support from others, no one can tell her exactly what to do.

For Hedda, her current being is at the same time familiar and unfamiliar. She is still on guard worrying about setbacks. She needs to experience a greater sense of familiarity if she is to gain a sense of belongingness in her being and be able to explore her possibilities in life. She must draw herself a new map in order to navigate a way home.

## Discussion

The two storylines presented here reveal being in recovery from ME/CFS as an inter-personal, contextual, fragile and nonlinear process in which the individual slowly and persistently builds a more homelike being, in both the body and the world. Recovery emerges as a continuously demanding process, one that requires determination, bodily based self-awareness and self-knowledge. In this discussion, we elaborate on this fragile process of (bodily) homecoming experienced by women recovering from ME/CFS.

As our participants reveal, the process of recovery entails searching for ways to make one’s being understandable and bearable again. After long periods in which they were bedridden and disconnected from the body and the world, the young women were in search of a new order. They searched for ways to gain bodily knowledge and trust; they strove to unite with a fragile body in a shared world of others. In line with Svenaeus (2011), this can be interpreted as a way of re-gaining belongingness by moving from an “unhomelike” being-in-the-world to one that is more “homelike” (Svenaeus, 2011). Rather than following a particular route it is an explorative process of searching for possible routes towards a new kind of homelikeness. For these young women, all of them living with a changed and changing body, the quest for homelikeness involved immense challenges.

Their fragile process of homecoming entailed searching and reaching for belongingness and reuniting with their bodily being. They embarked on a long and strenuous journey, a struggle to make sense of their strange body and how it reacted to movements and stimuli such as bright light and sound. Moving forward, exposing themselves to new experiences they regained a sense of belongingness in their being-in-the world. New possibilities and hope increasingly came in the foreground of their focus. But they also suffered moments of doubt—setbacks that could leave them feeling lost again and losing hope. Throughout this journey, the women sought to re-establish a kind of equilibrium and harmony in their being-in-the-world (Gadamer, 1996/2018).

Trying and failing formed part of this process of exploring the body during recovery. As they explored, the young women learned more about their bodies and bodily limits. This bodily exploration required strong self-motivation and a readiness to take risks: there was always a possibility of becoming worse, but also an opportunity of rising again, even if this involved great effort. Additionally, this bodily exploration entailed gaining knowledge and learning from setbacks. As the women persisted their exploring, past experiences of their bodies gradually faded away; they gradually regained trust in a body which, aided by growing self-understanding, was on course to become a new, habitual one. However, the fragility of this process was very much present in most of the stories, with the threat of relapse lingering in the background.

Over time, everyday routines and actions in the world gradually became pre-reflective: products of habit to which the women did not consciously pay attention (Merleau-Ponty, 1962). At the same time, the “new” habitual body called for attention in the sense that the women needed to be on guard for bodily signs and reactions. They sought to pick up on signs of exhaustion or other indications that they had pushed themselves too much. The new habitual body could manage to stay in balance and avoid setbacks when specific adjustments were made, new routines established, and some degree of balance achieved in respect of sleep, rest and meals. These specific and individual adjustments were entailed balancing on a thin line and emerges as the very essence of the recovery process in ME/CFS. In recovery, developing bodily self-knowledge emerges as an ongoing process of exploration, a constant effort to make sense of bodily limits and possibilities. This effort to make changes in one’s life and focus on possibilities has also been highlighted in previous research (Kalla & Simmons, 2020).

For our participants, a crucial moment in their recovery was their decision to reveal to others the extent of their illness. Through such self-exposure, the women were able to share common experiences, support one another, enrich their understanding, and create a sense of belongingness (Leder, 1990). Through others we widen our perspective on the world and in this way supplement our own embodiment (Merleau-Ponty, 1962). When we are in recovery, the experience of a fragile body may seem less alienating when shared with others who have similar experiences, as narrated in the first storyline. During recovery, shared experiences can contribute to our gaining knowledge about our being-in-the-world and make it less uncanny. The metaphor “letting go of the mask” describes this risky moment of revealing oneself as ill to self and others. Even though there is an aspect of existential loneliness in the experience of being severely ill, experiences of body and self, have a profoundly social dimension, one arising out of the presence of others and their gaze back upon us. As Leder puts it, “My self-understanding always involves the seeing of what others see in me” (Leder, 1990, p. 96). Exposing to others the extent of one’s illness may result in acceptance or rejection. But more importantly a step has been taken towards being honest with oneself, to being accepted by oneself without “the mask”.

Some participants gradually found themselves with sufficient energy to perform simple tasks, such as knitting, watching a film, or drawing (parents often played an encouraging role here). For others, their recovery experience was more of a sudden waking up. Whatever its form, this bodily awakening can be likened to being brought back, or reconnected, to life, to feeling the presence of life in a shared world of others. Uncertainty persisted. Participants were aware that their attempts to reconnect with body and outside world might fail. As narrated by the participants, these awakening experiences can be understood as “healing dramas” (Mattingly & Lawlor, 2001). As described by Mattingly (2010), small events or healing dramas can bring hope and therefore be of great importance. For our participants, new possibilities for action were opened up and new meanings arose as events unfolded (Mattingly & Lawlor, 2001). At the same time, these small events and dramas might be fleeting and fragile, their outcome uncertain. One participant used the metaphor “like rising from the grave” to describe her bodily awakening. The metaphor conveys not just the act of resurrection but also the unhomelike nature of the place from which one is rising: the bleakness of the grave. In the awakening storyline, there are glimpses of hope, of escape from the uncanny, unhomelike being in one’s own body and world, which has permeated every aspect of the individual’s being. There is the prospect of moving, of inching towards a more homelike being-in-the-world—one that is different from that experienced prior to falling ill.

During recovery, participants struggled to make sense of setbacks, with some experiencing a kind of collapse. Lack of comprehension and coherence, combined with difficulty in finding the words to describe such experiences to others, could undermine their attempts to build greater self-understanding. In order to cope with such reversals, which one participant likened “ending up in the ditch”, the need is to re-establish equilibrium, to regain balance in one’s being (Gadamer, 1996/2018/2018).

During the fragile recovery process described in the storylines, both illness and wellness seemed to be present. The daily need to make adjustments might help explain why for participants found it difficult to describe themselves as recovered from ME/CFS (Cheshire et al., 2020). Adults’ experiences of feeling healthy but at the same time adjusting to illness are described by Brown et al. (2017) as akin to living in a kind of borderland between illness and wellness: a threshold or liminality which could be understood as even less legitimate than actually being ill (Brown et al., 2017). This constant adjusting is in line with Paterson’s (2003) Shifting Perspectives Model of Chronic Illness. Here, Paterson argues that living with chronic illness involves an ongoing shift in perspective between illness-in-the-foreground and wellness-in-the-foreground, with the two perspectives co-existing and serving specific functions in the person’s world. This ongoing shift between wellness and illness is also described by Frank (1995). When being in recovery or member of “the Remission society”, there is a shift when wellness is in the foreground and illness in the background and the other way around depending upon the circumstances (Frank, 1995). For the participants in our study, it was crucial to create a feeling of certainty, increase their sense of control, and make sense of setbacks while seeking to avoid them. It was important that a sense of wellness prevailed over a sense of still being ill, even when they were still suffering from a disease.

Gadamer (1996/2018/2018), too, describes the importance of working one’s way back from the social disruptions caused by illness and returning to life, with all its challenges. The existential loneliness experienced by the participants while they were bedridden, isolated and detached from life contrasts sharply with their being in the process of recovery. Rather than being simply a matter of physical recovery from illness, this involves returning to where we belong, to where we truly live our lives (Gadamer, 1996/2018/2018). For our participants, the fact that many years had passed since they had last taken part in social activities, together with their restored ability to be part of activities associated with a healthy life, encouraged them towards a greater focus on wellness.

The fragile process of moving from an unhomelike being-in-the-world towards a new, different homelike being-in-the-world seems to rest on hard-fought-for knowledge about one’s body and its new ways of relating and acting. The body slowly becomes a new-habitual body, making it possible for the individual to carry out new habitual actions. During this inter-personal, contextual, fragile and nonlinear homecoming, illness slowly fades into the background and there is the prospect of a healthier tomorrow.

### Methodological considerations

In terms of robustness and trustworthiness, the findings of this study are reinforced by the transparency of the analysis and also by the fact that the authors have different professional backgrounds and scientific experiences, enabling us to challenge and question each other’s preunderstandings and interpretations. The collaboration between the authors allowed us to reflect and discuss across narrative methods and phenomenological perspectives. The authors brought different preunderstandings regarding ME/CFS treatments and the concept of recovery, resulting in much discussion, clarification and raising of critical questions. Throughout the research process we sought to maintain a reflexive, open and curious attitude while remaining critically conscious of our preunderstandings (Finlay, 2008). Throughout the process of analysing and writing, we benefited from discussions within the group. For instance, when analysing the stories, especially those involving “awakenings”, it was not always easy to understand what the recovery process was about. But through repeated reading and group discussions, we were able to gain a better grasp of the “awakenings” phenomenon and its nuances. What we first coded differently, eventually seemed to revolve around the same phenomenon.

The participating young women formed a relatively heterogeneous sample, drawn from different parts of Norway and with varying family backgrounds and educational levels. We considered the heterogeneous sample a strength, given that it yielded a variety of recovery stories and meaning-making strategies. The stories narrated by our participants share common features, underlining their relevance for a broader understanding of the experience of being in recovery. But despite the similarities across our interviews, our study does not claim to have revealed all possible recovery narratives; indeed, such a claim would be inconsistent with the narrative approach we have employed. However, we would argue that our study is an important contribution to understanding the effortful, complex, and fluctuating healing process undertaken to recover from ME/CFS.

## Clinical implications

This study highlights young women’s recovery from ME/CFS as a continuously demanding process that requires an explicit focus on bodily based self-awareness, self-knowledge and self-motivation. Our findings underline the importance of listening to, and learning from, the stories of women who are in recovery or have recovered from ME/CFS, if we are to gain richer, more nuanced understanding of what recovery involves and demands. In addition, our research suggests that it is vital for individuals in recovery to make sense of setbacks along the way. It is crucial to underscore the fact that the process of recovery usually takes years, with unavoidable setbacks along the way. Explaining the time dimension may help those in recovery by underlining the value of patience, openness to learning, and determination never to give up. That healthcare professionals should convey this message to patients striving to recover from ME/CFS seems of paramount importance. Healthcare professionals also need to remain aware of the possible discrepancy between their own understanding of what recovery entails and the perspectives and insights of their patients. Acknowledging this discrepancy, health care professionals can take more active part in their patients’ explorative, meaning-making process towards a new kind of homelikeness, thereby easing their challenges and burdens somewhat.

## Concluding remarks

Recovering from ME/CFS emerges as an inter-personal, contextual, fragile and nonlinear homecoming, based on a gradual rising bodily based self-knowledge. Crucial to such recovery is an individual’s ability to reach a state of being in which wellness predominates over illness. Recovery seems to be an ascending process, one in which illness slowly fades into the background as the healing patient moves on and looks ahead towards a healthier tomorrow.

In terms of further research, the authors highlight the need for more exploration of the specific health needs of children and adolescents in recovery from ME/CFS as they navigate a path to their own homecoming.
